# Supplementary material for: Assessing the climate benefits of afforestation in the Canadian Northern Boreal and Southern Arctic
Source: Nat Commun. 2025 Feb 25;16:1964. doi: 10.1038/s41467-025-56699-9 (PMC11861297; doi:10.1038/s41467-025-56699-9)
Supplement: Supplementary file 1 — Supplementary Information [file 41467_2025_56699_MOESM1_ESM.pdf]

Supplementary

Uncertainties in albedo-related afforestation assessments

Research has recently expanded beyond carbon sequestration, acknowledging changes in albedo due to varying tree cover [1-5], suggesting that many global biomes may exhibit a significant albedo offset, rendering afforestation climate negative. Forests modify a land cover's albedo by reflecting less shortwave radiation, retaining more energy on the ground, and majorly influencing surface temperature. The claim that forests are beneficial for carbon sequestration but detrimental to albedo, and therefore climate benefits should be evaluated based on the trade-off between the two, appears straightforward at first. However, this claim is overly simplistic to be useful in any practical setting for several reasons. First, forests do not alter carbon and albedo alone, but are involved in several Earth system processes (permafrost dynamics, non-radiative processes, aerosol forcing, hydrological processes, snow cover dynamics), none of which can be satisfactorily ignored. Second, the methodology employed in most albedo studies has major limitations that can reverse their conclusions (neglect of soil organic carbon (SOC) and greenhouse gas (GHG) emissions change, inadequate characterization of forest structure, limitations of remote sensing products, ignoring temporal and seasonal analysis, not considering planned nature of afforestation). Third, afforestation is a local endeavor influenced by regional realities and landscape-level details, which global remote sensing analyses cannot capture. Fourth, forests will be significantly impacted by the changing climate, and any afforestation analysis that neglects this is incomplete. Moreover, these studies overlook the complex interplay between disturbance-based mortality, albedo, and carbon uptake, which is a crucial consideration in understanding the effectiveness of afforestation efforts. Finally, considering only the positive feedback from albedo, tree growth in the southern arctic would theoretically lead to a runaway forest expansion due to reduced albedo increasing temperatures. However, there is no evidence from Earth's climatic history to support this claim, suggesting that important corrective mechanisms are being ignored in studies that focus solely on albedo.

In addition to the above arguments, the methodological uncertainties in albedo-related afforestation studies pertaining to remote sensing products, climate models, and radiative forcing (RF) kernels are discussed in more detail below.

Remote sensing products

It's important to acknowledge the uncertainties in remote sensing products used in albedo-related afforestation studies [1, 3]. For example, uncertainties in satellite-derived albedo can be as high as 9.7 W/m^2 [29]. While studies using remote sensing data to investigate the effects of land cover on albedo [1, 3] employ high-spatial-resolution albedo land use maps (LUMs) [6], they ultimately analyze data in monthly aggregates, which can mask important details. Temporal resolution significantly impacts final results [7], and this is particularly evident when examining the variability of albedo climatology (see Fig. 1). In regions with distinct snow and non-snow seasons, such as the boreal and tundra, coarser resolutions may fail to capture dynamic snow cover and clearing on vegetation [7]. Notably, the 8-day aggregate shows an albedo variability of less than 0.1, while the monthly variability exceeds 0.2 in boreal regions [7] (see Supplementary Fig. 1). This highlights the need to analyze albedo data at finer temporal resolutions than monthly aggregates.

Remote sensing data has limitations, including its inability to accurately estimate surface albedo and temperature under overcast conditions with cloud cover [8, 9]. Additionally, remote sensing albedo

products are susceptible to bias when the solar zenith angle (SZA) exceeds 70°, which is particularly relevant at high latitudes during boreal winter [10]. Furthermore, satellite-derived albedo products lack the spatial resolution to account for finer variations in topography, which affects solar radiation intensity, illumination angles, and snow cover [1, 11-13]. To ensure accurate region-specific albedo offset studies, it is essential to address the significant uncertainties related to overcast conditions, SZA, and topography.

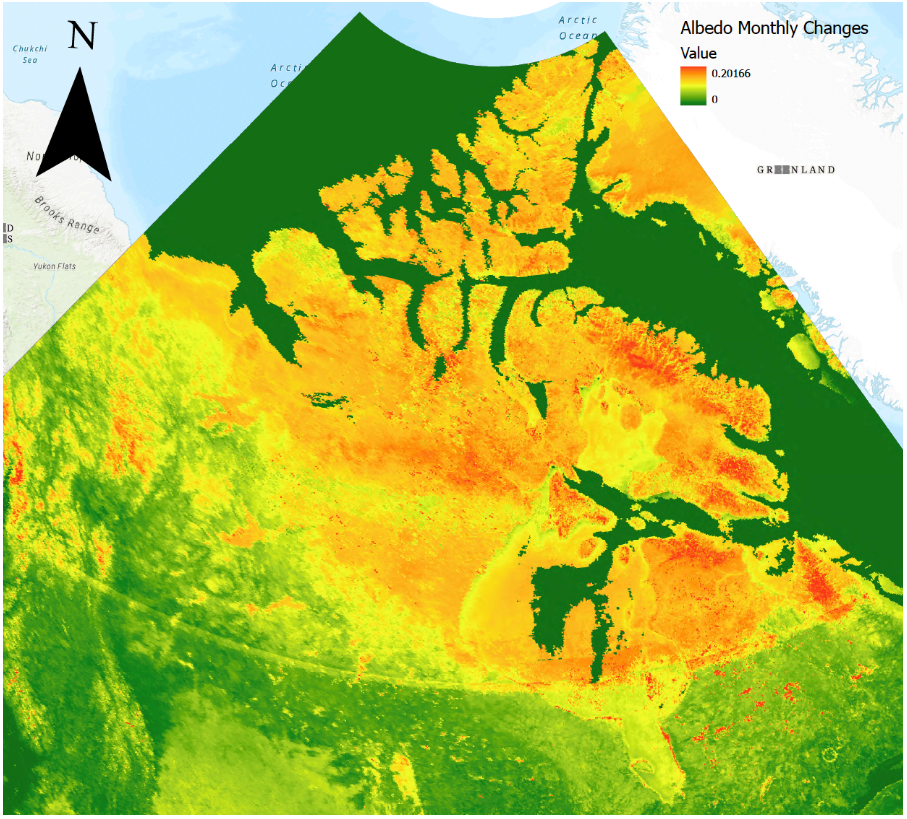

Supplementary Figure 1: Average standard deviation of monthly moderate resolution imaging spectroradiometer (MODIS) albedo data. The daily post-processed 500-m global surface blue-sky albedo climatology data is obtained at 0.05° resolution [7]. The daily data is aggregated to monthly standard deviations, and the 12-month average of the monthly standard deviations is computed. Many parts of the boreal have a high standard deviation of 0.1-0.2, because of the distinct snow season and lack of temporal resolution to capture the dynamic interaction between snow and vegetation.

Another source of uncertainty arises from land cover classification, both in the original satellite data and post-processing methods. For instance, the monthly moderate resolution imaging spectroradiometer (MODIS) land cover product often misclassifies savannas as forests and patchy forests as savannas [1]. In such cases, studies typically adopt a conservative approach regarding albedo offset, which can bias the final conclusions [1, 3]. Additionally, when sufficient pixel data is lacking, studies often rely on average data for the ecoregion using neighborhood analyses [1]. However, this approach can lead to spatially coarse and biased results. For example, some studies assume the potential end-state land cover for most of the boreal region to be either woody savanna or evergreen needleleaf forests [1] (see Supplementary Fig. 3 for current land cover [14]). This overlooks the significant presence of deciduous

trees, such as trembling aspen, which according to the Canadian National Forest Inventory (NFI) [15] comprise approximately 40% of boreal plains, 13% of taiga plains, and 14% of boreal shield. Given that deciduous forests have a lower albedo offset than evergreen ones due to their structure and dynamics [7], this could lead to a considerable underestimation of albedo values in the boreal region.

Reconciliation with climate models

While remote sensing observations offer flexibility in spatio-temporal modeling and global scale analysis, reconciling them with climate model simulations is crucial due to the models' ability to connect observed variables with various Earth system processes, including biogeochemical (BGC), biogeophysical (BGP), and hydrological processes. Discrepancies between remote sensing observations and climate model simulations indicate either incomplete process representations in models or artifacts in observational data. One such discrepancy is the albedo bias in climate models, which can lead to significant uncertainties in snow albedo feedback (SAF), affecting surface energy balance, temperature, and snow behavior [10] (see Supplementary Fig. 2). This albedo bias exhibits regional patterns, with climate models overestimating observations in boreal regions (positive bias) and underestimating observations in arctic regions (negative bias), likely due to premature snowmelt [16].

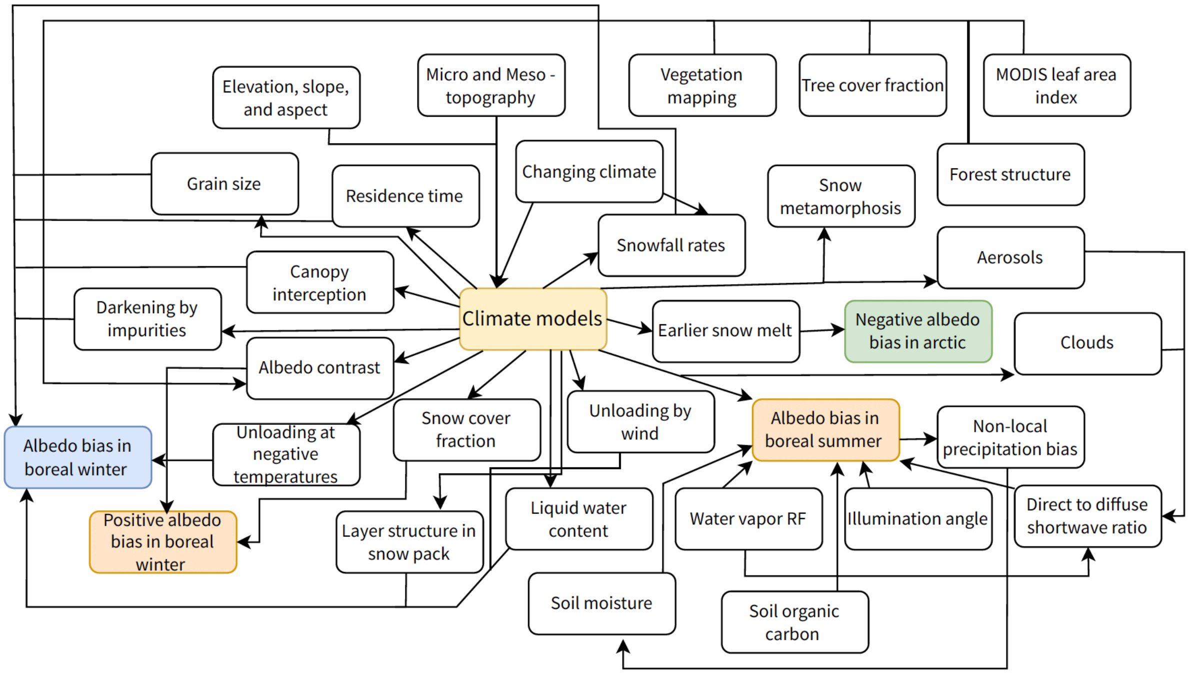

Supplementary Figure 2: A graph showing the various ways in which albedo bias manifests in climate models. Climate models have insufficient representations of canopy interception, unloading, residence time, darkening by impurities, and snowpack structure and content, which lead to albedo bias in boreal winter. The two major sources of error are the values of snow cover fraction (SCF) and albedo contrast, which contribute to a positive albedo bias in winter. While a modeling of earlier snowmelt might lead to negative albedo bias in the arctic, the bias during the boreal summer could be due to uncertainty in a variety of factors like water vapor RF, soil moisture, SOC, illumination angles, and direct to diffuse shortwave ratio.

Snow is the primary contributor to model biases in boreal regions, due to its high albedo and the lack of consistent measurements for model evaluation. Climate models' representations of snow are associated with multiple sources of uncertainty (see Supplementary Fig. 2), including: a) snow cover fraction (SCF), b) snow-covered albedo values, c) snow masking by boreal forests, d) canopy snow interception and residence time, e) canopy snow removal at negative temperatures, f) snow unloading by wind at the canopy top, g) effects of snow grain size, h) darkening by snow impurities like black carbon and dust, i) snowpack structure and water content, j) snowfall rates, and k) mechanisms of snow metamorphosis. Research has shown that the multi-model Coupled Model Intercomparison Project Phase 5 (CMIP5) mean albedo significantly differs from the MODIS albedo during boreal winter [10, 17]. These differences can be attributed to SCF errors at lower latitudes and albedo contrast (relative albedo weighting of SCF) at higher latitudes [10, 18]. While models' representations of canopy interception of snow and snow masking effects of forests have been shown to be unrealistic, the impact of other snow-related representations on the albedo bias remains unclear (see e-k above) [17, 19].

Climate models' higher snow-covered albedo values during boreal winter are partly due to inaccurate representations of vegetation mapping, forest structure, tree cover fraction, and leaf area index (LAI) [10, 18] (see Supplementary Fig. 2). Models with lower LAI values predict higher albedo over boreal forests. However, this is somewhat circular, as climate models calibrate using MODIS-derived LAI, which is unusually low over boreal forests during winter [10]. This discrepancy highlights the issue of uncertain ground truth when field measurements are lacking. Relying solely on MODIS SCF and albedo without reconciling with climate models leaves us vulnerable to other artifacts in MODIS products. While correcting the albedo bias in climate models may seem straightforward, modifying snow-covered albedo values could introduce numerous biases in critical processes like radiative flux, energy flux, and precipitation [10, 19].

Although smaller than the bias during boreal winter, the albedo bias during summer has a greater impact on energy fluxes that influence precipitation, due to higher solar radiation [16, 20]. Research has shown that the correlation between albedo and precipitation bias in CMIP5 models during boreal summer is causal, meaning that albedo bias leads to non-local precipitation bias, not vice versa [18, 20]. For instance, studies have found a negative correlation between terrestrial precipitation and summer albedo, potentially due to either brighter land reducing precipitation or wetter land having lower albedo [20] (see Supplementary Fig. 2). In addition to snow and vegetation-related factors, the summertime albedo bias may be caused by atmospheric constituents like water vapor that absorb shortwave radiation [18-20]. Other contributing factors could include illumination angle and the ratio of direct and diffuse shortwave radiation, which is affected by scattering from atmospheric constituents like water vapor, aerosols, and clouds [17, 18]. Finally, inconsistent modeling of soil moisture and soil organic carbon (SOC) may also contribute to the albedo bias [20] (see Supplementary Fig. 2).

Given that albedo interacts with and is impacted by numerous Earth processes, it is crucial to reconcile albedo observations from remote sensing with climate models before drawing definitive conclusions about afforestation based solely on albedo's negative effects. This approach ensures that we do not solely consider the negative effects of albedo in isolation but rather take into account its broader context within the Earth system.

RF kernels

The conversion of surface albedo to top-of-atmosphere (TOA) albedo relies on RF kernels, which, despite being used in ensemble predictions, introduce an uncertainty of approximately 15% in TOA conversions [1, 21, 22], which is even more pronounced in the boreal and tundra biomes. Additionally, the relationship between afforestation and clouds is insufficiently characterized in this conversion. Clouds significantly impact the quantification of albedo, as the conversion of surface albedo to TOA albedo relies on vertical profiles of atmospheric optical characteristics, which are influenced by cloud cover [23]. Clouds reduce the surface albedo contribution to TOA albedo through atmospheric attenuation of outgoing radiation, affecting the magnitude of RF associated with afforestation [23]. Furthermore, studies overlook the fact that RF from CO_2 and albedo influence different vertical structures, with a similar RF from these two agents resulting in different alterations to surface temperature [23]. To accurately model this effect, it is essential to consider different climate sensitivities before comparing the two.

Acronyms and abbreviations

Acronym/ Abbreviation	Expansion	Definition
BGC	Biogeochemical	Related to the transformation and movement of chemical elements and compounds between the Earth, atmosphere, and organisms. Key BGC processes involve elements such as carbon, water, phosphorus, nitrogen, and sulfur, which cycle through ecosystems, sustaining life and regulating Earth's climate and natural systems.
BGP	Biogeophysical	The combination of physical, biological, and geological processes acting in a region such as land surface roughness, albedo, and evapotranspiration.
GHG	Greenhouse Gas	Atmospheric gases that increase the surface temperature of planets like Earth. What sets them apart from other gases is their ability to absorb the radiation emitted by the planet, which leads to the greenhouse effect.
IPCC	Intergovernmental Panel on Climate Change	United Nations intergovernmental body responsible for advancing scientific understanding of climate change resulting from human activities.
SLCF	Short-lived Climate Forcer	A group of physically and chemically reactive compounds with atmospheric lifetimes generally less than two decades. This group includes particulate matter (PM), aerosols, and reactive gases like methane, ozone, and volatile organic compounds, among others.
SOC	Soil Organic Carbon	This carbon is the primary component of Soil Organic Matter (SOM), which consists of organic residues in

		different stages of decomposition within the soil. SOC represents the largest carbon reservoir on land, holding more carbon than the combined total in the atmosphere and vegetation.
ET	Evapotranspiration	Combined processes through which water moves from the Earth's surface to the atmosphere, encompassing both evaporation of water and plant transpiration.
mPWP	Mid-Pliocene Warm Period	A time period within the Pliocene (3.3–3.0 million years ago), when atmospheric CO_2 levels were comparable to those of today, and global temperatures were ~3 °C warmer than pre-industrial times.
ka BP	Thousand Years Before Present	A time scale primarily used in archaeology, geology, and related scientific fields to indicate when events occurred in relation to the advent of practical radiocarbon dating in the 1950s.
HTM	Holocene Thermal Maximum	This warm period occurred during the first half of the Holocene epoch, roughly between 9,500 and 5,500 years BP, with the highest temperatures, or thermal maximum, occurring around 8,000 years BP.
SST	Sea Surface Temperature	The temperature of the ocean's surface waters, measured using sensors on satellites, ocean reference stations, ships, and buoys. Since the ocean covers a large percent of the Earth's surface, scientists monitor SST to better understand the interactions between the ocean and Earth's atmosphere.
RF	Radiative Forcing	It is a concept in climate science used to measure the change in Earth's atmospheric energy balance. This change is influenced by factors such as greenhouse gas concentrations, surface albedo variations, aerosols and shifts in solar irradiance.
PAR	Photosynthetically Active Radiation	This is the range of solar radiation between 400 and 700 nanometers that photosynthetic organisms can utilize for the process of photosynthesis.
NPP	Net Primary Productivity	This is the amount of carbon generated by primary producers per unit time and area. It is calculated by subtracting plant respiration from total photosynthesis.
NEP	Net Ecosystem Productivity	It represents the carbon produced by plants through photosynthesis that is not respired by plants themselves, and heterotrophs.

PFT	Plant Functional Type	It is a classification system used by scientists to group plant species based on their similar roles and behaviors within an ecosystem.
BVOC	Biogenic Volatile Organic Compound	These compounds produced by plants play essential roles in plant growth. They are crucial in biosphere–atmosphere interactions and significantly influence the chemical and physical properties of the atmosphere.
SOA	Secondary Organic Aerosol	Molecules formed through the oxidation of a parent organic molecule over several generations. Unlike primary organic aerosols, which are directly emitted from the biosphere, SOAs are generated either through progressive oxidation of organic compounds or by condensing onto pre-existing particles.
ERF	Effective Radiative Forcing	ERF includes both the instantaneous forcing and the subsequent adjustments from the atmosphere and surface. It is a crucial metric for assessing the impact of human activities and natural factors on the climate.
TOA	Top-of-Atmosphere	It plays a crucial role in Earth's energy budget, where solar energy enters the Earth system, and reflected light, along with invisible thermal radiation from the Sun-warmed surface, exits. The balance between incoming and outgoing energy at the top of the atmosphere dictates Earth's average temperature.
ABL	Atmospheric Boundary Layer	It is the part of the troposphere that is directly affected by Earth's surface and responds to surface influences within an hour or less.
LST	Land Surface Temperature	It is a key variable in the Earth's climate system. It reflects processes like the exchange of water and energy between the atmosphere and land surface.
SZA	Solar Zenith Angle	It is the angle between the sun's rays and the vertical direction, representing the sun's position relative to the zenith.
MODIS	Moderate Resolution Imaging Spectroradiometer	It is an instrument that gathers remotely sensed data, which scientists use to monitor, model, and evaluate the impacts of natural processes and human activities on the Earth's surface.
CERES	Clouds and the Earth's Radiant Energy	They are instruments part of NASA's Earth Observing System (EOS) which are designed to measure both

	System	solar-reflected and Earth-emitted radiation, from the top of the atmosphere (TOA) down to Earth's surface.
HWP	Harvested Wood Products	They are materials derived from forests, used to create items such as paper, plywood, furniture, or utilized for energy.
SDG	Sustainable Development Goals	Adopted by the United Nations in 2015, these goals serve as a universal call to action to protect the planet, and ensure that all people experience peace and prosperity. The 17 Sustainable Development Goals (SDGs) are interconnected, acknowledging that progress in one area impacts outcomes in others, and that development must harmonize economic, environmental, and social sustainability.

Supplementary Table 1: Acronyms and abbreviations. Acronyms and abbreviations used in the manuscript along with their expansions, definitions, and importance.

Figures

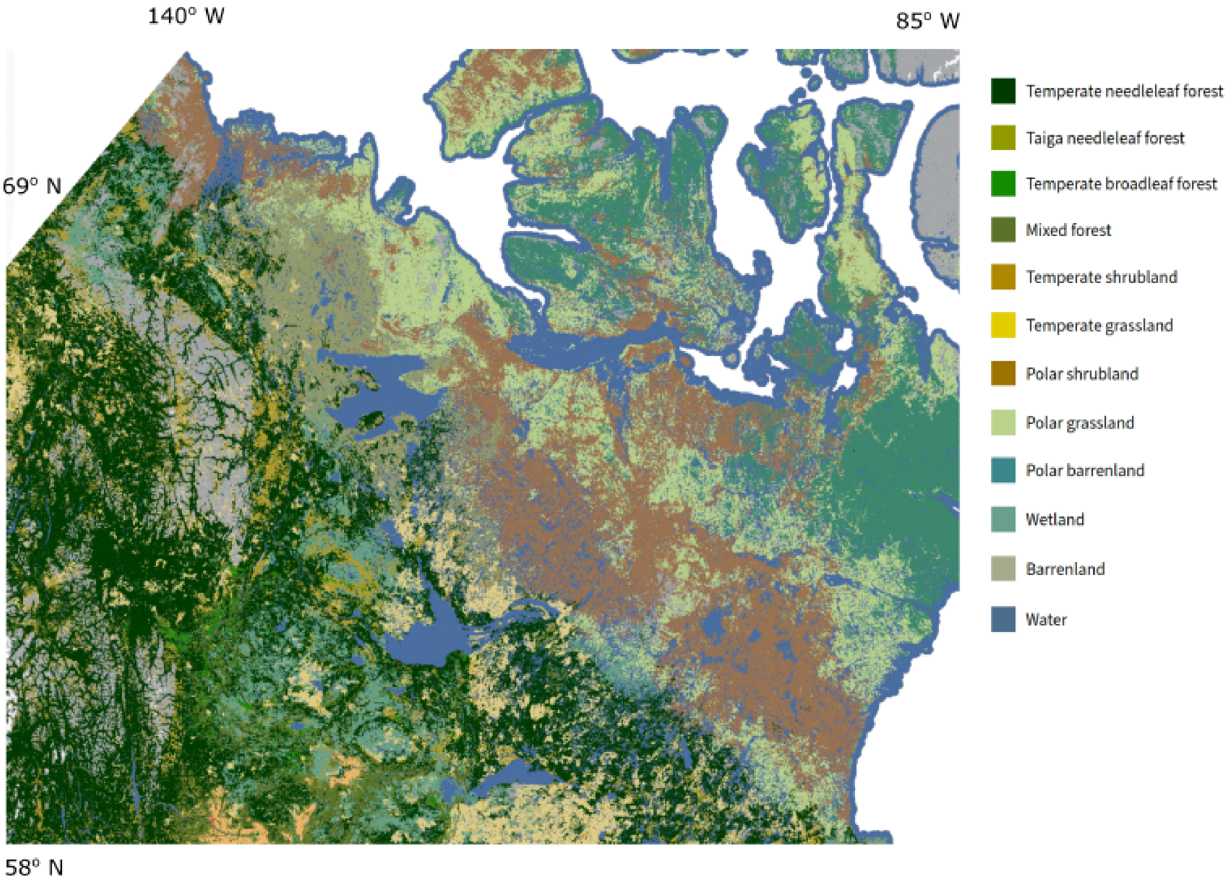

Supplementary Figure 3: Land cover classification for north-western boreal. Data obtained from [14]. The north-western boreal is predominantly made of temperate and taiga needleleaf (evergreen) forests, but also has a significant percentage of temperate broadleaf (deciduous) and mixed forests. In order to determine which species would grow best in the gaps between forested regions and surrounding non-forested regions, the tradeoffs between different components in a) need to be considered.

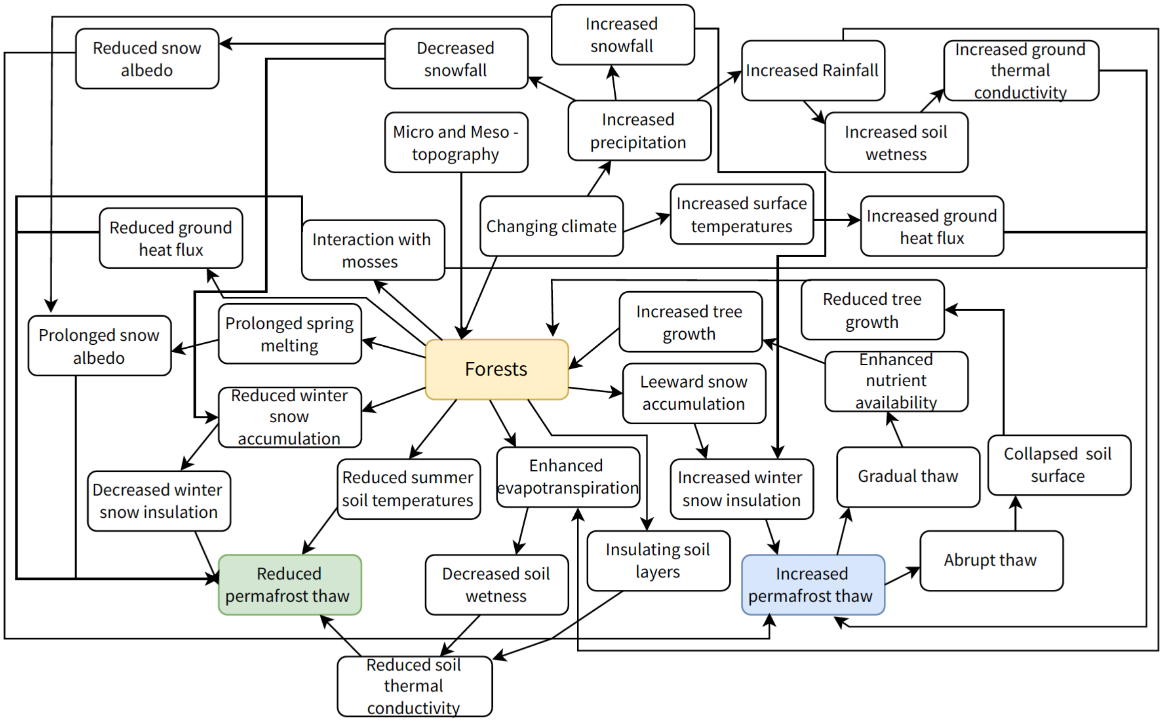

Supplementary Figure 4: A graph representing the dependencies between forests, permafrost dynamics, and thawing regimes. Forests reduce summer soil temperatures and winter snow accumulation, thereby reducing permafrost thaw. The enhanced evapotranspiration (ET) reduces soil wetness, therefore the soil thermal conductivity, preserving permafrost. The efficient energy redistribution in forests reduces ground heat flux and the prolonged spring melting increases the snow-related albedo. Moreover, the interaction between forests, mosses, and insulating soil layers play a key role in maintaining permafrost stability. Gradual thaw and abrupt thaw have different implications for vegetation productivity, however, both thawing regimes are expected to worsen because of changing climate.

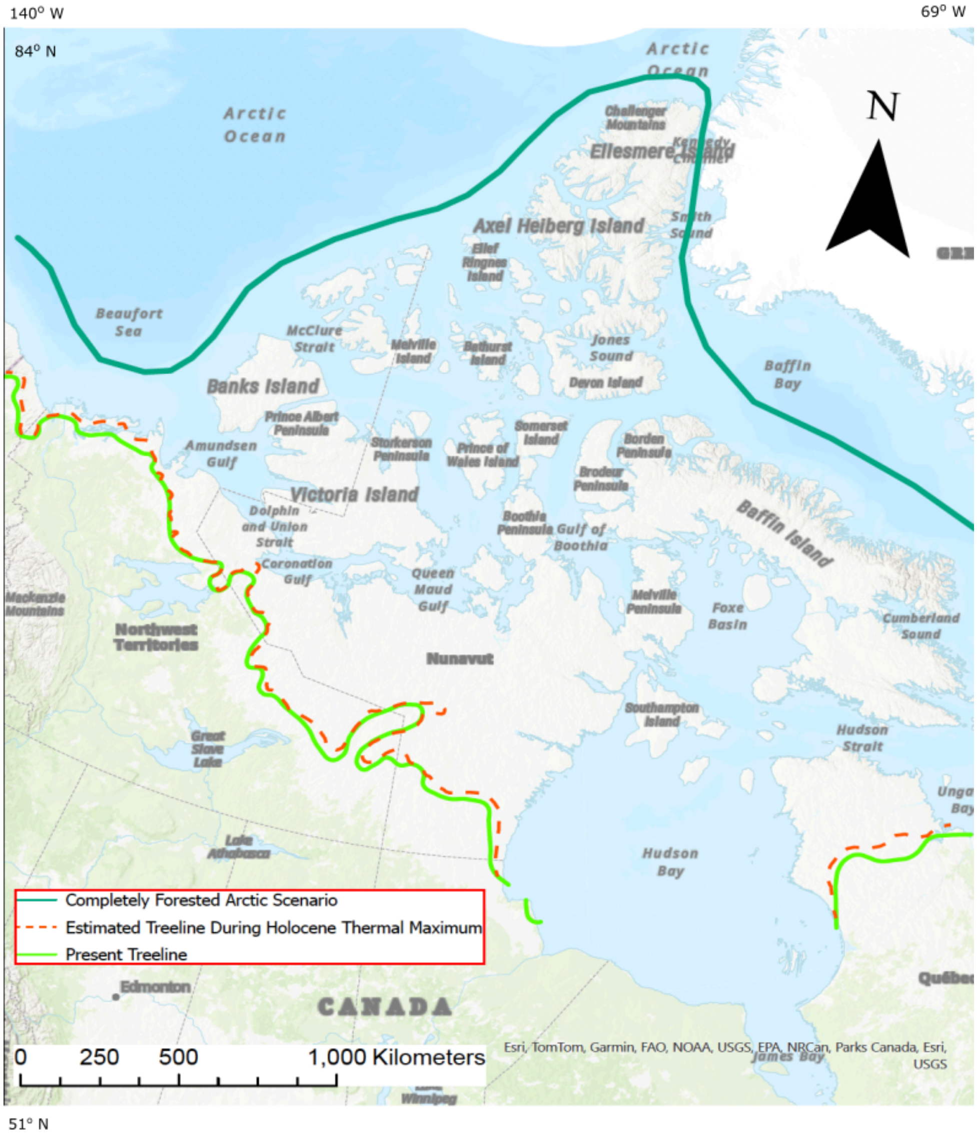

Supplementary Figure 5: Tree line extents during present day and altered climate states. A climate state in which the boreal fully expands into the arctic does not reach equilibrium and is pushed back to a climate state with an extended tree line that might have existed during the Holocene thermal maximum (HTM), suggesting that the warming from overall climate feedbacks is insufficient to push the boreal forest from the HTM tree line to complete Arctic forestation.

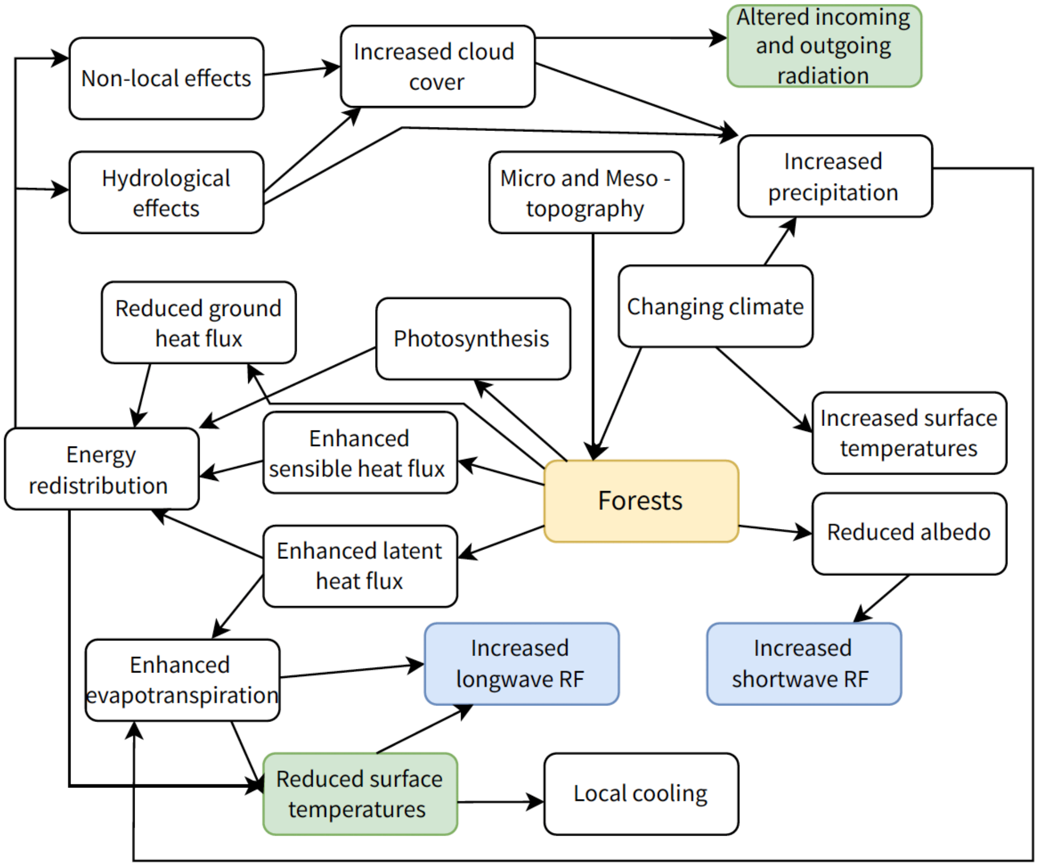

Supplementary Figure 6: A graph showing how forests affect radiative and non-radiative processes, altering shortwave and longwave RFs, surface temperatures, and net radiation balance. Forests distribute the incoming energy into processes like photosynthesis, sensible heat flux, and latent heat flux. The enhanced ET from increased latent flux reduces surface temperatures. This contributes to local cooling but also induces longwave RF. While the reduced albedo in forests induces shortwave RF, the efficient energy redistribution affects non-local and hydrological processes, which in turn alter atmospheric energy balance. Non-radiative processes are expected to dominate in a changing climate, as temperature and precipitation are key drivers.

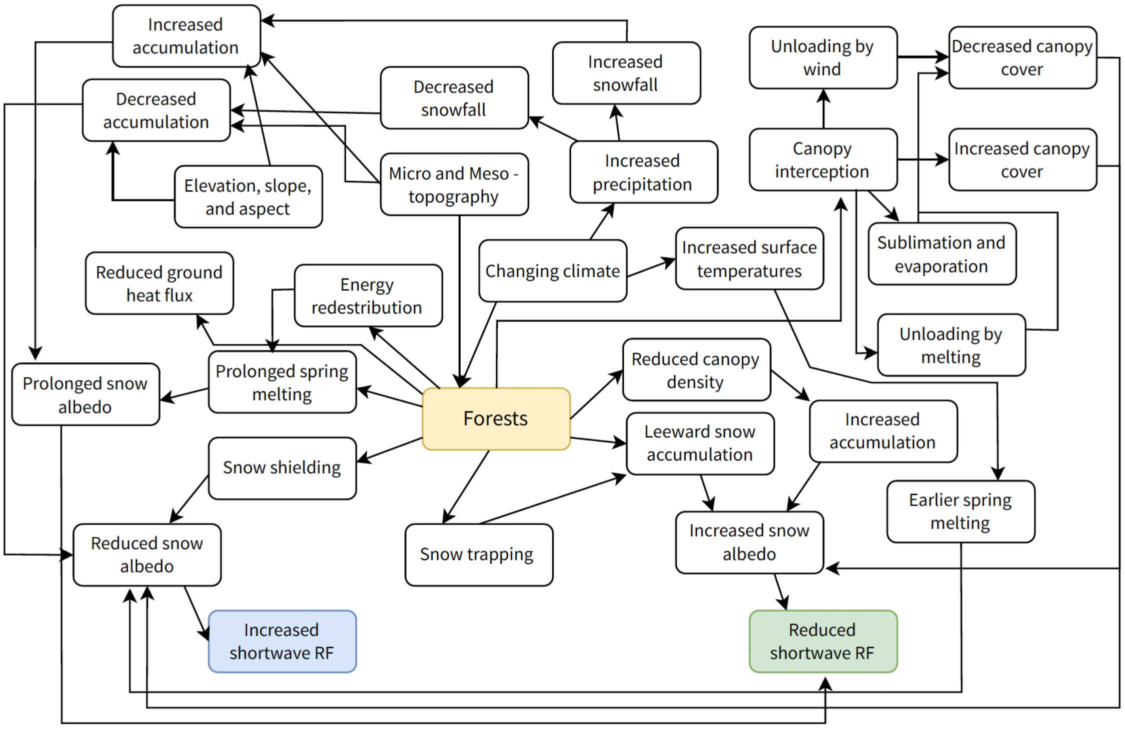

Supplementary Figure 7: A graph depicting the interaction between forests and snow, changing energy and radiation balance at the surface. Forests with dense canopies shield snow on the ground, thereby reducing snow albedo. On the other hand, both leeward snow accumulation and prolonged spring melting can increase the snow albedo. Forests with reduced canopy density like deciduous forests increase the snow albedo by reducing the snow shielding effect. Efficient canopy interception and resistance to unloading by dense canopies can theoretically increase forest albedo, however, more measurements are needed to confirm this observation. A changing climate will alter the precipitation rates as well as the melting regimes, thereby changing the forest-snow interaction further.

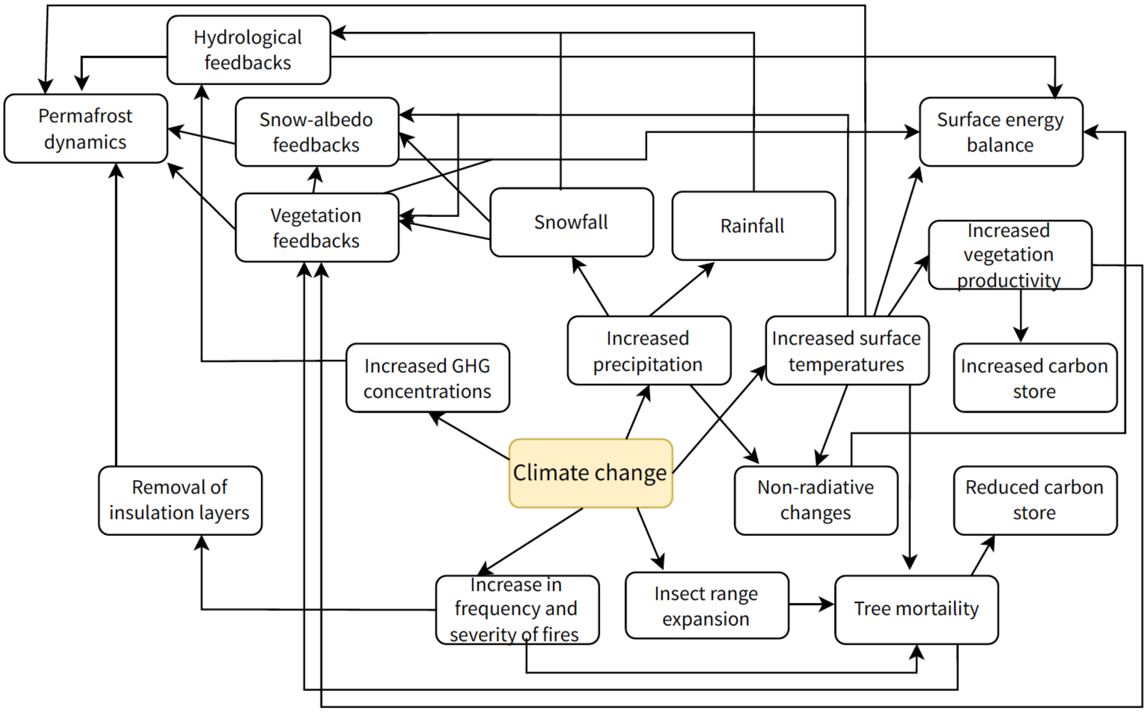

Supplementary Figure 8: A graph depicting the interactions between forests and a changing climate. Climate change is projected to increase the severity and frequency of forest fires, causing tree mortality and removal of soil insulation layers that protect permafrost. Insect range expansion is also estimated to increase tree mortality and reduce carbon stores. While the increased temperatures might increase vegetation productivity at high latitudes, the increased precipitation is going to alter surface energy balance, and hydrological and albedo feedbacks.

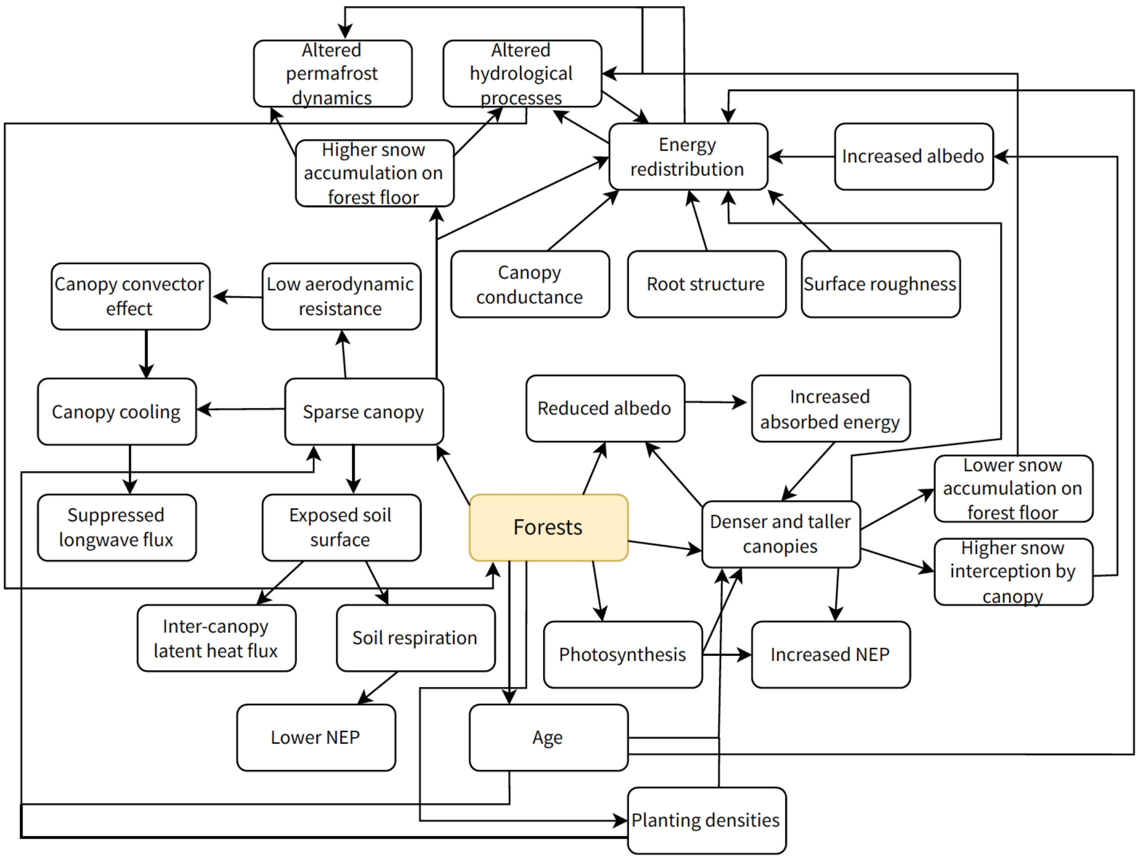

Supplementary Figure 9: A graph showing the relationship between forest structure and energy balance. The absorbed energy by forests creates denser and taller canopies, which in turn increases absorbed energy. Denser canopies have lower snow accumulation on the forest floor and higher interception at the top of the canopy, both of which affect forest energy balance. Sparse canopies contribute to canopy cooling, but also increase longwave RF and soil respiration. Sparse canopies also have higher snow accumulation on the forest floor, reducing albedo, and altering permafrost and hydrological dynamics. The difference in forest structure as trees age also dictates canopy-snow interception and energy redistribution.

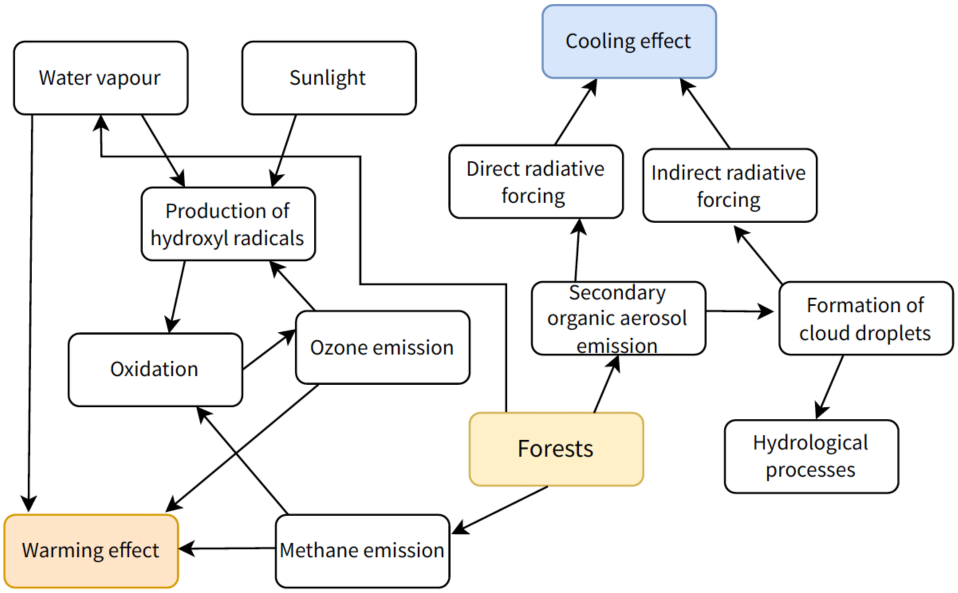

Supplementary Figure 10: A graph showing the effects associated with BVOC emissions by forests. Forest BVOC emissions alter methane, water vapor, and SOA concentrations. Water vapor produces hydroxyl radicals in the presence of sunlight which react with methane to form ozone. Both methane and ozone cause a warming effect. On the other hand, the SOA particles produce a cooling effect, either through DRF or IRF via cloud formation.

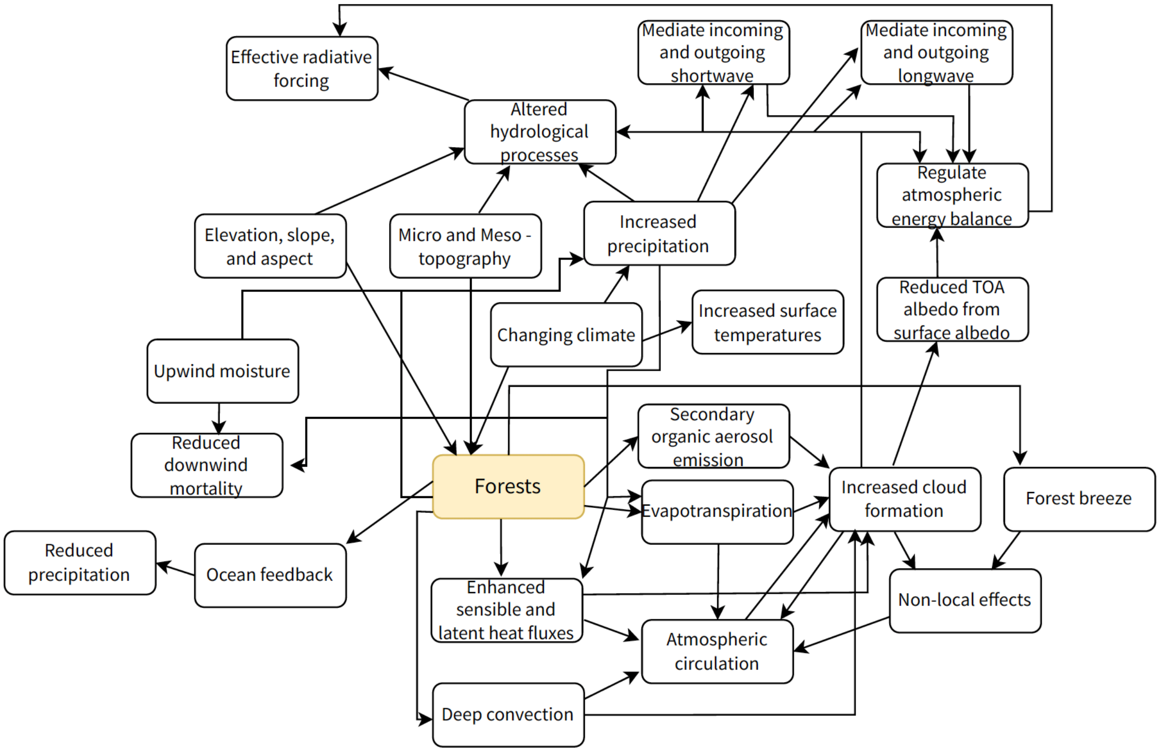

Supplementary Figure 11: A graph depicting how forests alter hydrological processes. Forests change atmospheric circulation through non-radiative processes, non-local effects, deep convection, and cloud formation. Cloud formation is itself affected by ET and secondary organic aerosol (SOA) emissions from forests. Clouds affect the earth's energy balance by mediating incoming and outgoing shortwave and longwave radiation, contributing to effective radiative forcing (ERF). Moreover, clouds alter the surface albedo contribution to TOA albedo. The increased temperature and precipitation with the changing climate are expected to alter hydrological processes significantly.

References:

1. Hasler, N. et al. Accounting for albedo change to identify climate-positive tree cover restoration. *Nat. Commun.* **15,** 2275 (2024).
2. Mykleby, P. M., Snyder, P. K. & Twine, T. E. Quantifying the trade‐off between carbon sequestration and albedo in midlatitude and high‐latitude North American forests. *Geophys. Res. Lett.* **44,** 2493-2501 (2017).
3. Jiao, T. et al. Global climate forcing from albedo change caused by large-scale deforestation and reforestation: quantification and attribution of geographic variation. *Clim. Change* **142,** 463-476 (2017).
4. Kirschbaum, M. U. F. et al. Implications of albedo changes following afforestation on the benefits of forests as carbon sinks. *Biogeosciences* **8,** 3687-3696 (2011).
5. Weber, J. et al. Chemistry-albedo feedbacks offset up to a third of forestation's CO_2 removal benefits. *Science* **383,** 860-864 (2024).
6. Gao, F. et al. Multiscale climatological albedo look-up maps derived from moderate resolution imaging spectroradiometer BRDF/albedo products. *J. Appl. Remote Sens.* **8,** 083532-083532 (2014).
7. Jia, A., Wang, D., Liang, S., Peng, J. & Yu, Y. Global daily actual and snow‐free blue‐sky land surface albedo climatology from 20‐year MODIS products. *J. Geophys. Res. Atmos.* **127,** e2021JD035987 (2022).
8. Windisch, M. G., Davin, E. L. & Seneviratne, S. I. Prioritizing forestation based on biogeochemical and local biogeophysical impacts. *Nat. Clim. Chang.* **11,** 867-871 (2021).
9. Bright, R. M. et al. Local temperature response to land cover and management change driven by non-radiative processes. *Nat. Clim. Chang.* **7,** 296-302 (2017).
10. Li, Y. et al. Evaluating biases in simulated land surface albedo from CMIP5 global climate models. *J. Geophys. Res. Atmos.* **121,** 6178-6190 (2016).
11. Hao, D., Wen, J., Xiao, Q. & Yu, W. Sensitivity of Vegetation Shortwave Albedo to Topography. In *IGARSS 2019-2019 IEEE International Geoscience and Remote Sensing Symposium* 6051-6054 (2019).
12. Klein, A. G., Hall, D. K. & Riggs, G. A. Improving snow cover mapping in forests through the use of a canopy reflectance model. *Hydrol. Process.* **12,** 1723-1744 (1998).
13. Raleigh, M. S. et al. Ground-based testing of MODIS fractional snow cover in subalpine meadows and forests of the Sierra Nevada. *Remote Sens. Environ.* **128,** 44-57 (2013).
14. Canada Centre for Remote Sensing, Canada Centre for Mapping and Earth Observation & Natural Resources Canada. 2020 Land Cover of Canada. *Government of Canada* https://open.canada.ca/data/en/dataset/ee1580ab-a23d-4f86-a09b-79763677eb47 (2022).
15. Hermosilla, T., Bastyr, A., Coops, N. C., White, J. C. & Wulder, M. A. Mapping the presence and distribution of tree species in Canada's forested ecosystems. *Remote Sens. Environ.* **282,** 113276 (2022).
16. Thackeray, C. W., Fletcher, C. G. & Derksen, C. Quantifying the skill of CMIP5 models in simulating seasonal albedo and snow cover evolution. *J. Geophys. Res. Atmos.* **120,** 5831-5849 (2015).
17. Wang, L. et al. Investigating the spread in surface albedo for snow‐covered forests in CMIP5 models. *J. Geophys. Res. Atmos.* **121,** 1104-1119 (2016).
18. Thackeray, C. W., Fletcher, C. G. & Derksen, C. Diagnosing the impacts of Northern Hemisphere surface albedo biases on simulated climate. *J. Clim.* **32,** 1777-1795 (2019).

19. Essery, R. Boreal forests and snow in climate models. *Hydrol. Process.* **12,** 1561-1567 (1998).
20. Levine, X. J. & Boos, W. R. Land surface albedo bias in climate models and its association with tropical rainfall. *Geophys. Res. Lett.* **44,** 6363-6372 (2017).
21. Forster, P. M. et al. Recommendations for diagnosing effective radiative forcing from climate models for CMIP6. *J. Geophys. Res. Atmos.* **121,** 12-460 (2016).
22. Smith, C. J. et al. Effective radiative forcing and adjustments in CMIP6 models. *Atmos. Chem. Phys.* **20,** 9591-9618 (2020).
23. Zhao, K. & Jackson, R. B. Biophysical forcings of land-use changes from potential forestry activities in North America. *Ecol. Monogr.* **84,** 329-353 (2014).
